# Between *simpatia* and *malandragem*: Brazilian *jeitinho* as an individual difference variable

**DOI:** 10.1371/journal.pone.0214929

**Published:** 2019-04-15

**Authors:** Marco Akira Miura, Ronaldo Pilati, Taciano Lemos Milfont, Maria Cristina Ferreira, Ronald Fischer

**Affiliations:** 1 Social Psychology Lab, Institute of Psychology, University of Brasília, Brasília, Brazil; 2 Centre for Applied Cross-Cultural Research, Victoria University of Wellington, Wellington, New Zealand; 3 Graduate Program of Psychology, Salgado de Oliveira University, Niterói, Brazil; 4 Instituto D’Or de Pesquisa e Ensino, São Paulo, Brazil; University of Lleida, SPAIN

## Abstract

Culture-specific behaviour strategies provide an interesting window into individual differences research, producing a richer conceptualization of personality descriptions. Our aim is to describe the personality dimensions linked to a core socio-cultural behaviour pattern in Brazil: *jeitinho*. To reach this goal we conducted four studies. Our first set of studies (1a, 1b and 1c) examined the underlying structure of *jeitinho* as an individual difference variable and its nomological network with social values, the Big-Five, moral attitudes, and social dominance orientation. In Study 2, we confirm this structure and relate personal *jeitinho* to perceptions of *jeitinho* norms. Results demonstrated that personal *jeitinho* has two dimensions: *Jeitinho Simpático* is an individual’s tendency to seek positive social interactions, avoid conflict, and find creative solutions; and *Jeitinho Malandro* captures behaviours such as the use of deception and trickery. These two behaviours are rooted in the same dimensions of the integrated model of values and personality.

## Introduction

Culture-specific behaviours provide an interesting window into personality expression and raise fascinating questions about human behavioural plasticity. Brazilian *jeitinho* (pronounced *jay-tchee-nyoo*) is one well-documented case, described as a ‘special way to solve a problem or a difficult or prohibited situation… [that involves] finding a creative solution for dealing with situations, whether in the form of conciliation, cunningness, or skill’ ([1] p.41). *Jeitinho* is a characteristic behavioural trademark of Brazilian culture, which explains why this phenomenon has received increasing attention from Brazilian social scientists [2–4] as well as international scholars [5–9]. Our study aims to describe *jeitinho* as a stable behavioural trait within a specific cultural context and link it to a network of established personality and individual difference dimensions that are near-universal, thereby contributing to a nuanced understanding of how (presumably) universal predispositions are displayed in adaptive ways within specific socio-cultural environments. Hence, our work extends and bridges work on culture-specific behavioural expressions and examines how consistent behavioural traits can fit into larger personality and individual difference frameworks.

Psychologists have started to define *jeitinho* core dimensions and related psychological processes, and have also compared it with other potentially related indigenous informal social influence processes in different cultures [5,6]. Previous studies primarily focused on normative processes and perceptions of cultural norms but did not focus on possible individual differences based on this informal social influence strategy [7,8,10]. Yet, individuals vary in the extent to which they engage in these behaviours, suggesting that there might be consistent individual differences in this culturally relevant behaviour [7]. In the present research, we focus specifically on these individual difference processes of *jeitinho*, mapping their structure and exploring the role of personality dimensions related to this behavioural trademark of Brazilian culture. If *jeitinho* is a broad set of culturally relevant behaviours in Brazilian culture, it is interesting to ask whether there are individual differences in self-reported behaviour that are influenced by the larger cultural script. Therefore, our study complements and extends previous studies by investigating the structure and correlates of individual differences centred around a complex socio-cultural behavioural strategy like *jeitinho*. This paper aims to explore personality dimensions related to socio-cultural behavioural norms, proposing an individual difference approach to the study of *jeitinho*. By focusing on the personality bases of individual differences in *jeitinho*, we contribute to the socio-relational and ethnopsychological approach to the study of personality [11], examining potentially universal personality dynamics through specific cultural expressions. Such specific cultural expressions cannot be captured by pan-cultural models of personality and might be described as a way to broaden our comprehension of individual differences bringing it closer to specific cultural factors that shape individuals’ expressions of personal characteristics.

A large body of literature suggests that there is a pan-cultural structure of personality in literate societies [11–13]. At the same time, a more culturally-oriented approach, or a combination of culture-specific and universal approaches, can produce a richer conceptualization of personality descriptions within specific cultural contexts [14–21]. The advantage of considering an emic perspective when developing personality instruments has been demonstrated in different cultural contexts. To cite a few recent examples, the South African Personality Inventory (SAPI) project has demonstrated the usefulness of combining an emic-etic approach in the development of culturally sensitive measures of individual differences, which capture unique characteristics of specific cultural groups while relating them to cross-culturally established models [19]. There are other research examples which seek to describe more culturally oriented personality descriptor in Mexico [22], China [23] and India [24].

These culture-specific personality studies have focused on personality as a way of capturing culturally relevant behavioural indicators for describing an individual across many social contexts and, at the same time, relate this culturally specific individual differences with pan-cultural personality models. This strategy is useful to improve the understanding of personality dimensions, describing the convergence and divergence of culturally-specific dimensions with broader personality models. In this paper, we used the same approach, focusing on a specific individual difference variable derived from a culturally relevant psycho-social construct but relating it to a broader model of personality and other individual difference indicators.

### *Jeitinho* core dimensions and its behavioural markers

The translation of the underlying term *jeito* (‘way’) characterizes the way people behave in social interactions in Brazil. *Jeitinho* as a cultural phenomenon has some core characteristics centred on notions of creativity, *simpatía* (affective and prosocial relationship orientation), and social norm-breaking and corruption [8]. Previous studies that described psychological underpinning of jeitinho defined the construct as a special strategy to solve problems, that involves a personal relationship and that uses cunningness, trickery, creativity and *simpatia* to achieve a purpose [7,8]. In a previous study, *jeitinho* was operationalized through behavioural scenarios that represent a person acting in a way that depict stereotypical forms of *jeitinho* [8]. For example, the corruption part was represented by a scenario such as: ‘Every time José takes a taxi for company purposes, he has the right to request reimbursement for the amount paid. When he is without money, he requests a receipt for a greater amount than he has paid and submits this to the company. He keeps the extra money’; whereas social norm breaking behaviors were measured by scenarios such as: ‘Parking at shopping centres is difficult during busy times. Knowing that it is very difficult to find a place to park at these times, Camila speaks to her grandmother and invites her to go shopping, so that she can park in a space reserved for the elderly’. ([7] p. 335). Brazilians recognize *jeitinho* as a common behaviour, but this complex set of characteristics can be evaluated both positively and negatively. The background of this behavioural syndrome is that Brazil is characterized by a restrictive and inflexible bureaucratic hierarchy combined with large income inequalities, both of which pervade many aspects of daily life and create obstacles and barriers for individuals [25]. In its most typical form, *jeitinho* involves a violation of social norms because individuals are restricted to pursue personal goals through these rigid social, economic and institutional constraints. In order to satisfy personal goals, individuals have to break social (or legal) norms, effectively creating a trade-off between the individual and the larger social and/or institutional interests. Not surprisingly, violating social and legal norms is evaluated negatively, explaining why many Brazilians evaluate *jeitinho* as a negative practice.

On the other hand, Brazilians are stereotypically known as cordial, kind, sympathetic, and affectionate people [1]. According to Triandis, Marín, Lisansky and Betancourt [26], *simpatía* is a cultural script typical of Hispanics and Latin Americans that reflects a general relationship-oriented pattern that includes (a) importance given to values of loyalty, respect, duty, and politeness, (b) emphasis on cooperation and interpersonal helping, and (c) willingness to sacrifice oneself for the sake of attending family functions. Individuals may spontaneously develop new relationships or draw upon existing relations to solve a problem. In addition to developing or using existing social relations, many Brazilians may use creativity to solve a personal problem. The use of social and creative skills is often positively evaluated by Brazilians, which explains the positive evaluation of some parts of *jeitinho*. These two core normative processes and perceptions of cultural norms around *jeitinho* (i.e., social or legal norm-breaking/deception and creativity/*simpatía*) are likely to form the main markers of individual differences in *jeitinho*.

How do Brazilians use *jeitinho-*related behaviours when describing themselves? As we noted above, it involves both positively as well as negatively valenced behaviours. Pilati et al. [8] identified a large number of behavioural strategies emerging from the analysis of critical incidences related to *jeitinho*. At a broader level, their categories differentiate between a relationship or ‘soft’ interpersonal component and a manipulative ‘dark’ component. Consequently, it is likely that individuals differentiate between dimensions that express *Jeitinho Simpático*, which is characterized by creativity and *simpatía*, and *Jeitinho Malandro*, which expresses deceptive and transgressive behaviours.

The first, *Jeitinho Simpático*, is the positively evaluated skill, which emphasises the creative and affectionate aspects of the strategy and represents an attempt to solve problems while also maintaining harmony in relationships within an excessively formal and bureaucratic environment. Behavioural markers of this dimension involve actions like (a) being polite with other people in different social environments; (b) acting in particular ways to establish personal contact with others; (c) behaving in ways which express kindness and care for others, even with unknown people and (d) finding creative ways to solve problems. In contrast, the second, *Jeitinho Malandro*, is negatively valenced because it involves a disregard for rules and may harm other people because an actor makes use of deception to take advantage of others. Behavioural markers of this dimension are (a) cunningness and deception in social relations with the purpose to reach a goal; (b) disregard of social rules and laws to gain personal advantage and (c) lying or deceiving other people.

### The present research

The present research is organized around two studies. The first study is organized into three sub-studies. The purpose of our first set of studies (Study 1a, 1b and 1c) was to examine the underlying structure of *jeitinho* trait descriptions (Study 1a) and to report correlations with personality traits, values, and moral and social attitudes (studies 1b and 1c).

Focusing on personality and values first, we used a version of the Big Five personality dimensions and personal values since both are an important aspect of personality that emerges through the interplay between genetic and environmental variables resulting in two broad behavioural dimensions that underlie both the Big Five and personal values [13]. The first dimension underlying both values and Big Five personality traits captures behavioural approach versus avoidance motivation which shares variance with both the value dimension Openness to Change and the Big Five traits Openness and Extraversion at one end; and behavioural avoidance motivation which shares variance with Conservatism values and Conscientiousness and Neuroticism Big Five personality trait factors. The second major dimension underlying both the Big Five and values captures different modes of cooperation, differentiating an instrumental orientation towards cooperation (with Self-Enhancement values and Extraversion and Conscientiousness Big Five traits loading on it) from a trust-based orientation to cooperation (which is reflected in Self-Transcendence values and Agreeableness Big Five traits). The relative strength of this association is affected by contextual threat [27]. These major dimensions of personality can be used to locate *jeitinho*-relevant traits in the larger personality domain. Previous research has shown that *jeitinho* is related to values and personality. Ferreira et al. reported that the Creativity dimension using behavioural scenarios was positively correlated with Agreeableness and Conscientiousness and Breaking Social Norms and Corruption behavioural scenario dimensions were negatively associated with Agreeableness and Conscientiousness. These previous results indicate the usefulness of analysing the relationship of personal *jeitinho* with personality variables and values, to contextualize this culture-specific behavioural trait within the context of broader individual differences within and across cultures [13].

In addition to these personality variables, *jeitinho* may show some meaningful relationships with social attitudes, especially attitudes related to social norms and morality. A previous study had suggested that perceptions of *jeitinho* are related to moral norms and social dominance orientation [7]. We, therefore, examine whether individual differences in social attitudes also correlate with personal *jeitinho*. Finally, in Study 2, we independently confirm the personal *jeitinho* structure and then relate self-reports back to perceptions of *jeitinho* norms [7].

## Study 1

Our first study tests the psychometric properties of personal *jeitinho*, and examines the nomological network of *jeitinho*. We administered our *jeitinho* measure to a large sample. Due to space restraints, we then administered different scales to subsets of the larger sample. Study 1a describes the development of the personal *jeitinho* measure and the structure in the overall sample. Study 1b reports data from a subset that assessed the associations between *jeitinho* and values, while Study 1c reports data from the subset which included a Big-Five measure, moral attitudes and social dominance orientation. All data analyses for study 1 were performed with SPSS v. 21.

### Study 1a

The main purpose of this study was to test the psychometric properties and factorial structure of the Personal *Jeitinho* Scale (PJS). Considering the structure and content of the *jeitinho* construct, our prediction was that the PJS items can be organised into at least two dimensions, one which will contain creativity and *simpatía* behaviours, and the other which will load items related to trickery and cunningness. More dimensions have been described in the literature using both ethnographic interviews and behavioural scenarios with provide more situational context [7, 8, 10]. Considering that we are developing a short individual difference measure, we would expect that at least these two core dimensions separating a positive and negative way of approaching problems may emerge.

#### Method

We collected data from 469 Brazilians (72.7% female, average age 34 years, SD = 12.47; age range 18–71) from all regions of Brazil, using an online survey. The study followed ethical guidelines of research with human subjects, informed consent was obtained, and all data were treated anonymously.

The development of the Personal *Jeitinho* Scale (PJS) was done based on a pool of 82 behavioural situations that was generated based on the critical incidents analysed by Pilati et al. [8]. The items were written by the first author in collaboration with the second author and two research assistants (undergraduate students in social psychology). The aim was to create short personality-like statements that capture the main behavioural dimensions that were identified in the previously published critical incidence analysis. We then assessed the relevance of these 82 statements for *jeitinho* by asking 13 judges (PhD and MSc students, staff members and advanced undergrad students in the social psychology programme) to rate the degree of clarity and relevance of the behavioural statement for describing Brazilian *jeitinho*. We also asked the judges to code them in accordance with the categories derived from Pilati et al [8]. A total of 37 behavioural situations were judged to clearly capture core aspects of *jeitinho* and to fall into only one of the core categories (‘harm to others’, ‘trickery’, ‘disregard for rules’, ‘*simpatía*’, and ‘creativity’). These situations were then used to write a pool of items describing specific actions or behaviours. This list of behavioural situations was presented to our participants who were asked to indicate the similarity of their own behaviour with the action described in each item on a 6-point scale (1-Does do not look like me; 6-Really looks like me).

#### Ethics statement

The studies reported in the manuscript strictly followed the Ethical Principles in the Conduct of Research with Human Participants proposed by the American Psychological Association. The participants were informed about the research purposes, the risks involved in taking part of the research, the confidentiality and anonymity of the participation. Participants explicitly informed, via oral communication (in the data collection done in classrooms) or checked a box (in the internet-based data collection), their consent in taking part in the research and were freedom to quit the participation in any moment. The registration of the consent in classroom data collection was registered by the completed questionnaire. No personal information was collected to preserve participants anonymity. The research protocol was not submitted to an approving institutional review board because the committee’s rules and regulations in Brazil only evaluate projects in the realm of medical and pharmacological research. Considering this situation in the current national regulations it is not required to obtain approval from an institutional review board to conduct studies with human subjects in social sciences.

#### Results and discussion for study 1a

A Principal Component Analysis identified 11 factors with eigenvalues greater than 1, explaining 53% of total variance. The screeplot suggested retaining two factors, which represented the most coherent theoretical representation of the internal structure of the items. Based on a principal factoring analysis, we eliminated items with a loading below a cut-off criterion of .30. Therefore, this final version consisted of 31 items loading on two factors. Table 1 shows the items’ factor loadings and reliability coefficients in relation to each factor based on the final data set of 31 items. These two factors together explained 15.5% of the total variance. The amount of explained variance of PJS and other measures used in our paper is similar to other cultural research. One issue with measuring broad cultural constructs is that a diverse set of behaviours are typically associated with these cultural phenomena. Hence, the bandwidth of relevant behaviours is broader and therefore, factor loadings and explained variance are lower. This trade-off between bandwidth and fidelity is common when measuring culturally relevant psychological constructs [28].

**Table 1:** Factor loadings final 31 items, and reliability coefficients of *jeitinho simpático* (JS) and *jeitinho malandro* (JM) factors.

Items		JS	JM
4	She/He likes to keep a pleasing social climate.	.60	
3	People feel valued and accepted when near her/him.	.61	
8	She/He offers help to coworkers.	.55	
30	She/He is creative when dealing with work challenges.	.51	
5	She/He holds the door open when someone else follows/approaches.	.49	
6	She/He always greets the doorman of her/his building by name every time she/he enters.	.44	
32	She/He seeks new opportunities for her/his career.	.44	
31	She/He always provides novel alternatives to figure out friends’ problems.	.43	
36	She/He invents new recipes when she/he has few food choices.	.40	
2	She/He greets unknown people when she/he walks on the street.	.40	
9	She/He measures words to avoid conflicts.	.35	
35	She/He orders different dishes every time she/he returns to the same restaurant.	.30	
29	She/He returns books to the library before the deadline.	.30	
7	She/He offers her/his house to be used for company celebrations.	.31	
17	She/He lies to obtain a goal.		.56
21	She/He, knowing that somebody will call in a specific time, turns off the mobile phone and says that the battery was over.		.50
18	She/He is tired on Monday and calls to her/his job saying is sick.		.50
11	She/He leaves the bar table without paying her/his part of the bill when everybody should share it.		.49
19	She/He enters in a party without a fee due to knowing the promoter.		.46
25	She/He follows the principle: ‘Rules were done to be broken’.		.45
24	She/He crosses the red light of traffic light when the street is empty and without traffic radar.		.45
20	She/He looks for an acquaintance who is a notary office employee to advance her/his request.		.43
16	She/He wants to buy new clothes to use in the weekend, but when she/he realizes the store is closing, convinces the saleswoman to still sell.		.42
28	She/He frequently does not pay the condominium fee on time.		.37
26	She/He throws trash on the floor.		.39
23	She/He parks in the disabled parking slot when the parking is full, and she/he is in a hurry.		.38
22	She/He uses a car without a seat belt when driving short distances.		.36
12	She/He speaks about the failures of other coworkers when chatting with her/his superiors.		.33
13	She/He seizes the opportunities to harm others.		.33
10	She/He double parks obstructing other vehicles.		.32
15	She/He chats during a movie session.		.32
	**Reliability coefficient (α)**	**.75**	**.77**

The first factor, called *Jeitinho Simpático*, had loadings from 14 items. These items focused on an individual’s tendency to seek positive social interactions, avoid conflict, and find creative and alternative solutions for solving problems. The second factor consists of 17 items and was called *Jeitinho Malandro*. This factor captures behaviours that include breaking or circumventing social rules and norms as well as using deception and trickery. This two-factor structure covers a broad theoretical description in anthropological work [1,25] and more recent psychological research [7,8].

### Study 1b

To understand the motivational structure of these two dimensions, we first focused on correlations with values as motivational goals [13,29]. Schwartz [29,30] proposes ten motivational value types that people use as guiding principles in their lives: Power, Achievement, Hedonism, Stimulation, Self-direction, Universalism, Benevolence, Conformity, Tradition, and Security. These ten values types are aggregated in two bidimensional continuum. The first Self-Transcendence (Universalism, Benevolence) to Self-Enhancement (Power, Achievement, Hedonism) and the second Openness to Change (Self-direction, Stimulation) to Conservation (Conformity, Tradition, Security).

Based on the ten value types described in the Schwartz model, we expect two different patterns of correlations with *Jeitinho Simpatico* and *Jeitinho Malandro*. The positive value factor of *jeitinho* is likely to correlate positively with Self-transcendence values as well as some Openness to change values. Benevolence values, in particular, express a concern with the well-being of others (especially those who are close to the actor). Similarly, Stimulation is related to seeking novelty, change and excitement and Self-Direction to a pursuit of intellectual endeavours and behaviours. We expected a positive correlation to the extent that there is a creativity component of *Jeitinho Simpático*. Taking into account the integrated model of values and personality by Fischer [13], *Jeitinho Simpático* is likely related to Behavioural Approach Motivation values that rely on trust-based cooperation.

With regards to the *Jeitinho Malandro* factor, our prediction is that Achievement, Hedonism, Conformity and Security might be correlated with this factor. In an earlier value study in Brazil [31,32], Tamayo included the value *esperteza* (or ‘cleverness’ but in a negative sense, is conceptually related to breaking of social norms, trickery and deception) and found that it related closely to Achievement values. It suggests that being *esperto* is seen as personal competence to achieve personal goals [31]. Hence, we could predict that Achievement will be positively correlated with *Jeitinho Malandro* type. Hedonism values might be positively related to *Jeitinho Malandro* because they express seeking pleasure and sensual gratification, which closely associated with the stereotype of being a *malandro* [33]. On the other hand, Conformity values might be negatively correlated with *Jeitinho Malandro* because behaving in a malandro way is a path to not conform with social standards. Finally, *Jeitinho Malandro* might be negatively correlated with Security values, considering it is a way to undermine the social fabric of society. For the integrated model of values and personality of Fischer [13], *Jeitinho Malandro* is negatively related to the Behavioural Approach and Trust-based cooperation, considering the pattern of correlations expected to happen.

#### Method

A subset of 210 participants (70.6% female, average age 33.8 years, SD = 12.3) from Study 1a completed the questionnaire version that included the values measure. An a priori sample size calculation determined a sample of 172 participants to detect a medium effect size, with an α of .05 and a power of .95.

**Personal values.** We used a Brazilian version of the Portrait Value Questionnaire [34], adapted by Tamayo and Porto [35], and measured on the same 6-point scale as the *jeitinho* measure. The Cronbach’s alphas varied from .38 (Tradition) to .81 (Hedonism). These coefficients are consistent with those previously observed in Brazilian samples [35].

#### Results and discussion for study 1b

Table 2 presents the results of bivariate correlations and a standard multiple regression analysis examining the relationship between value types and the two dimensions of personal *jeitinho* factors. As predicted, Benevolence and Stimulation were the main statistical predictors of *Jeitinho Simpático*. Benevolence expresses motivational values that show care for the well-being of others, which is an important mechanism through which *simpatía* is expressed. Stimulation refers to motives related to novelty-seeking and change, which underpin the creativity component of this personal *jeitinho* factor. Self-direction also statistically predicted this *jeitinho* factor. This can be explained because Self-Direction values relate to motives such as independence of action, which is relevant to the creativity component of *jeitinho*.

**Table 2:** Summary of simple regression analyses and bivariate correlation for *jeitinho simpático* (JS) and *jeitinho malandro* (JM) with the ten motivational types of schwartz values model as antecedents (N = 210). 95% confidence interval for B are in brackets.

													*Model I* *JS*	*Model II* JM
Variable	Stim	SelfDi	Univ	Ben	Conf	Trad	Sec	Pow	Ach	Hed	JS	JM	*β* *[B 95%CI]*	*β* *[B 95%CI]*
Stim	-												.21* [.03;.23]	-.001 [-.11;.09]
SelfDi	.54+	-											.16* [.02;.28]	.06 [-.08;.18]
Univ	.24+	.26+	-										.12 [-.02;.23]	-.10 [-.21;.04]
Ben	.27+	.24+	.48+	-									.25+ [.10;.33]	.06 [-.06;.17]
Conf	-.05	.07	.35+	.33+	-								.07 [-.04;.17]	-.29+ [-.32;-.11]
Trad	-.19+	-.06	.28+	.18+	.43+	-							.03 [-.08;.11]	.19* [.03;.22]
Sec	.11	.28+	.37+	.28+	.46+	.30+	-						.08 [-.05;.17]	-.24* [-.28;-.06]
Pow	.23+	.26+	-.07	.05	-.05	-.13	.11	-					-.04 [-.10;.06]	.14 [-.01;.15]
Ach	.17*	.23+	-.02	.07	.02	-.20+	.13	.61+	-				-.07 [-.13;.05]	.08 [-.05;.13]
Hed	.41+	.12	.14*	.16*	-.07	-.07	.13	.24+	.37+	-			.03 [-.06;.09]	.23* [.04;.19]
JS	.38+	.37+	.40+	.45+	.25+	.13	.29+	.02	.01	.15*	-			
JM	.11	.02	-.17*	-.05	-.35+	-.09	-.26+	.23+	.19+	.26+	-.05	-		
Mean	4.1	4.9	5.0	5.1	4.5	3.8	4.6	3.3	4.1	4.3	4.4	1.9		
SD	.95	.68	.69	.72	.80	.87	.78	1.15	1.08	1.13	.61	.59		
*R*^*2*^													.34 .31	.27 .23
*Adj R*^*2*^														
*F*													10.49**	7.52**

*p < .05.

+*p* < .01.

Stim = Stimulation; SelfDi = Self-direction; Univ = Universalism; Ben = Benevolence; Conf = Conformity; Trad = Tradition; Sec = Security; Pow = Power; Ach = Achievement; Hed = Hedonism; JS = *Jeitinho Simpático*; JM = *Jeitinho Malandro*

For *Jeitinho Malandro*, as predicted, Hedonism was positively correlated. Hedonism is relevant because using *Jeitinho Malandro* is a way of reaching sensual gratification. As can be seen in Table 2, Tradition appears as a significant statistical predictor in the regression analysis, but the analysis of bivariate correlation shows that it is a suppression effect, due the high correlations between a number of the predictor variables. As predicted, Conformity and Security negatively related to this *jeitinho* factor. *Jeitinho* expresses social norm-breaking and threatens social integrity which explains the negative relationship between Conformity and Security values with *Jeitinho Malandro*. The expected relationship with Achievement values was not supported. Perhaps the value of *esperteza*, investigated by Tamayo and Schwartz [31], is a more abstract representation of *jeitinho* than the behavioural descriptions that we used here. Further investigations should explore this issue.

In summary, *Jeitinho Simpático* is more strongly related to Self-Transcendence and Openness to Change values, *Jeitinho Malandro* is strongly negatively related to Conservation values. Together, these results support the idea that there are consistent motives that underlie personal *jeitinho*, possibly linked to the behavioural approach versus avoidance motivations [13].

### Study 1c

Study 1b examined the correlation of personal *jeitinho* with Big Five traits, morality and social dominance orientation. *Jeitinho Simpático* involves using social skills, having interpersonal sensitivity, and a high level of sociality, which points towards Agreeableness as a correlate of it [36,37]. In addition, Openness to Experiences may correlate with this *jeitinho* component because it is related to how individuals may seek new ways of solving problems [38], which is expressed in the creativity aspect of *Jeitinho Simpático*. For the *Jeitinho Malandro* dimension, we predict that Conscientiousness will be negatively correlated because high levels of Conscientiousness are associated with norm-abiding orientation.

We also included a measure of social and moral attitudes. First, a previous study by Ferreira et al. [7] found that individuals with strong moral attitudes towards not breaking social and legal norms are less likely to engage in *jeitinho* behaviours. Those who strongly internalize moral norms about upholding rules and regulations are less likely to report engaging in *Jeitinho Malandro* dimension. We do not expect a strong correlation with the *simpatia* component because this dimension does not violate social or moral sentiments, but also does not reinforce them. Finally, individuals who accept that some social groups are superior to other social groups (Social Dominance Orientation) [39] in life might be more callous in pursuing a strategy that advances their own interests (see Ferreira et al.). Specifically, we expected a positive correlation effect only for the *Jeitinho Malandro* dimension, since individuals who endorse this dimension are more prone to justifying social norm-breaking as a natural way of social hierarchies.

#### Method

A subset of 196 participants (73.8% female, average age 34.2 years, SD = 12.7) from Study 1a completed the questionnaire version that included the personality traits and social and moral attitudes. An a priori sample size calculation determined a sample of 160 participants to detect a medium effect size, with an α of .05 and a power of .95.

**Big-Five Personality Inventory.** We used the shortened 20 item version of the Big-Five personality inventory [38], which has been adapted for a Brazilian context by Gouveia, Meira, Santos, Jesus, and Formiga [40]. This measure is a shortened version of BFI [41] with short phrases to assess the most prototypical traits of Big Five dimensions (sample items: is talkative; is depressed, blue; has a forgiving nature). The inventory had acceptable Cronbach’s alpha reliability coefficients: .80 (Openness to New Experiences), .68 (Conscientiousness), .77 (Extraversion), .60 (Agreeableness), and .79 (Neuroticism).

**Moral attitudes.** We used a 4-item shortened version of the morally debatable behaviour scale (MDBS) utilized in the World Values Survey (WVS, see [42]). Our shortened version evaluated moral issues only related to dishonest-illegal behaviours, such as not paying taxes or accepting bribery. Participants were asked to indicate on a 10-point scale (1-Never justified; 10-Always justified) how morally justifiable they rated each behaviour. Cronbach’s alpha for this scale was .63.

**Social Dominance Orientation (SDO).** A balanced six-item version of the SDO scale was used [39]. We considered the new two-factor conceptualization of SDO formed by a dominance (SDO-D) and an anti-egalitarianism (SDO-E) factor [43]. These factors had acceptable Cronbach’s alpha for a short measure: .53 and .59, respectively.

#### Results and discussion for study 1c

To test our predictions of the relationship between personality variables and personal *jeitinho*, we conducted two hierarchical regression analyses for each of the personal *jeitinho* dimensions entering SDO in step 1, moral attitudes in step 2 and the Big-Five dimensions at the final step (see Table 3). This order was chosen to evaluate the effect of attitudes before personality factors. SDO-D negatively correlated with *Jeitinho Simpático* but this association vanished when including the personality traits. As predicted, both Openness to Experiences and Agreeableness positively correlated with this *jeitinho* factor, and the final model explained close to 30% of the variability in *jeitinho*. As predicted both Agreeableness (because of the underlying social orientation) and Openness (because of the creativity component) were correlated with greater *Jeitinho Simpático*. These results corroborate our predictions based on Fischer [13], suggesting that this type of *jeitinho* behaviour is driven by the Behavioural Approach and Trust-Based cooperation dimensions.

**Table 3:** Summary of hierarchical regression analysis and bivariate correlations for SDO, moral attitudes and big-five factors predicting both personal *jeitinho* factors (N = 196). 95% confidence interval for *B* are in brackets.

											*Model I JS*			*Model II JM*		
											Step 1	Step 2	Step 3	Step 1	Step 2	Step 3
Variable	SDO-D	SDO-E	*M*	Op	Con	Ext	Agr	Neu	*JS*	*JM*	*β* *[B 95%CI]*	*β* *[B 95%CI]*	*β* *[B 95%CI]*	*β* *[B 95%CI]*	*β* *[B 95%CI]*	*β* *[B 95%CI]*
SDO-D	-										-.24+ [-.18;-.05]	-.24+ [-.18;-.05]	-.13 [-.12;.002]	.09 [-.02;.10]	.04 [-.04;.08]	.003 [-.05;.06]
SDO-E	-.16	-									-.05 [-.09;.04]	-.04 [-.09;.04]	-.02 [-.07;.05]	-.01 [-.07;.04]	-.02 [-.07;.04]	.001 [-.06;.05]
M	.13	.02	-									-.03 [-.07;.04]	.02 [-.04;.06]		.30+ [.06;.15]	.26+ [.04;.13]
Op	-.13	-.04	-.01	-									.23+ [.06;.23]			.01 [-.07;.09]
Con	-.09	.14*	-.14*	.25+	-								.05 [-.07;.14]			-.26+ [-.28;-.09]
Ext	-.11	-.04	-.14*	.44+	.38+	-							.11 [-.02;.15]			.04 [-.05;.11]
Agr	-.23+	.06	-.14	.25+	.27+	.44+	-						.24+ [.08;.31]			-.12 [-.18;.03]
Neu	.12	.14	.06	-.06	.04	-.05	-.21+	-					-.12 [-.11;.006]			.09 [-.01;.09]
JS	-.24+	-.02	-.04	.38+	.21+	.35+	.41+	-.20+	-							
JM	.10	-.03	.34+	-.06	-.32+	-.14	-.22+	.13	.04	-						
Mean	2.43	5.09	2.23	5.57	6.02	5.44	5.94	4.53	4.46	1.94						
SD	1.28	1.33	1.55	.98	.78	1.08	.77	1.39	.61	.53						
*R*^*2*^											.05	.06	.29	.01	.10	.19
*F* Δ *R*^*2*^												.20	12.00+		19.32+	4.51*

*p < .05.

+*p* < .01

SDO-D = Dominance; SDO-E = Anti-egalitarianism; M = Moral; Op = Openness; Con = Conscientiousness; Ext = Extraversion; Agr = Agreeableness; Neu = Neuroticism; JS = *Jeitinho Simpático;* JM *= Jeitinho Malandro*

*Jeitinho Malandro* was correlated with moral attitudes: people who are more socially lenient tend to describe themselves as more of a *Jeitinho Malandro* type. This result is in line with previous research [7]. We also found a negative relationship with Conscientiousness, which is in line with the theoretical predictions, suggesting that more conscientious individuals are less likely to report social norm-breaking behaviours. This suggests that *Jeitinho Malandro* motivations are also based in Behavioural Approach, but with an instrumental control orientation [13].

## Study 2

We have identified two broad dimensions underlying an individual difference measure of *jeitinho*, which relate systematically to values, behaviours and moral attitudes. In our second study, we aimed to replicate this structure and then relate it to previously reported *jeitinho* normative processes [7] in a different sample. Specifically, we predict individual differences in *Jeitinho Simpático* will be positively correlated with the Creativity-related scenarios, whereas *Jeitinho Malandro* will be positively related to both perceptions of Corruption and Social Norm-Breaking *jeitinho* scenarios.

### Method

The sample was composed of 284 undergraduate students from Brasília, most of whom were female (N = 216), with a mean age of 26 years (SD = 9.77). To test the regression model an a priori sample size calculation determined a sample of 119 participants to detect a medium effect size, with an α of .05 and a power of .95.

#### Personal *Jeitinho* Scale

We used a short version of the scale used in Study 1. We selected 18 items; nine for each factor, including only items with factor loadings above .40 (see Table 1). We also changed the response scale to have seven points (1-Does do not look like me; 7-Really looks like me).

#### Brazilian *Jeitinho* Questionnaire

This questionnaire consists of 21 scenarios that describe an actor solving a problem using three forms of *jeitinho*. The first factor, Creativity, describes situations in which the main actors use creative solutions to solve a problem without violating a social or legal norm. The second factor, Corruption, consists of scenarios in which the problem-solving strategy involves illicit means. The third factor, Social Norm Breaking, focuses on strategies that bypass social norms in order to solve a problem. The reliability indices are similar to those reported by Ferreira et al. [7]: Creativity α = .52; Corruption α = .78; Social Norm-Breaking α = .75.

### Results and discussion for study 2

Our first goal was to test the two-factor structure of the PJS observed in Study 1a. We ran a confirmatory factor analysis using Maximum Likelihood estimation in Amos v. 21. The model tested was a latent factor model with two correlated latent variables, one variable to *Jeitinho Simpático* and other to *Jeitinho Malandro*. The initial fit was less than perfect: χ^2^(76) = 251.41; χ^2^/df = 1.90; CFI = .81; RMSEA = .057 (CI90% = .046, .067). The correlation between the two latent variables was close to zero (Φ = .021; p = .477). Modification indices suggested that the fit would improve with the elimination of items 11, 31 and 36 (see Table 1). The goodness-of-fit indices after removing these three items, using only the remaining 15 items were adequate: χ^2^(87) = 138.96; χ^2^/df = 1.60; CFI = .90; RMSEA = .046 (CI90% = .031, .060)). The reliability coefficients were similar to Study 1a: *Jeitinho Simpático*, α = .63, ω = .66; *Jeitinho Malandro*, α = .71; ω = .72. These results indicate that the short version of the PJS had acceptable psychometric properties and replicated the structure found in study 1.

We then performed two regression models with the observed scores of each *jeitinho* factor, testing how individual differences relate to *jeitinho* (see Table 4). This analysis was conducted with SPSS v. 21. As predicted, only the Creativity scenarios emerged as significantly correlated with *Jeitinho Simpático*. For *Jeitinho Malandro* the Social Norm-Breaking scenarios were significantly correlated with this dimension of personal *jeitinho*, partially corroborating our prediction. The absence of correlation of our new measure with Corruption scenarios may be due to the absence of clear identification of illegal actions that violate laws in our personality-like measure.

**Table 4:** Regression models for *jeitinho simpático* (JS) and *jeitinho malandro* (JM) factors with BJQ personal factors as predictors. 95% confidence interval for *B* are in brackets.

						Model I *Jeitinho Simpático* (JS)	Model II *Jeitinho Malandro* (JM)
Variable	*Creativity*	*Corruption*	*SNB*	*JS*	*JM*	*β* *[B 95%CI]*	*β* *[B 95%CI]*
Creativity (BJQ)	-					.32+ [.11;.23]	.02 [-.06;.09]
Corruption (BJQ)	.18*	-				-.03 [-.08;.05]	.07 [-.03;.12]
Social Norm Breaking (BJQ)	.26+	.56+	-			-.12 [-.09;.008]	.51+ [.30;.55]
*Jeitinho Simpático (*JS)	.28+	-.04	-.05	-			
*Jeitinho Malandro* (JM)	.18*	.35+	.56+	.01	-		
Mean	6.38	1.67	4.32	5.64	2.73		
SD	1.64	1.83	2.36	.87	1.15		
*R*^*2*^						.10 .09	.31 .30
*Adj R*^*2*^							
*F*						10.02+	42.0+

*p < .05.

+*p* < .01.

## General discussion

Stable individual differences can only be understood if seen through the lens of the demands and constraints of the specific cultural and economic context [44]. If personality researchers only look for pan-cultural expressions of personality independent of culturally specific expressions, they may miss important variation of human behaviour as well as indices of behavioural plasticity. One useful path is to examine how socio-cultural behaviours fit within well-established individual difference frameworks. In our work we developed a new individual measure that captures one well-described cultural characteristic within the Brazilian context and then examined the plausible underlying personality dimensions related to this cultural problem-solving strategy. Although based in a specific cultural context, the main argument is that stable individual differences in culturally normative behaviours can be psychologically studied and related to broader personality and attitudinal systems. Hence, our study maps out emic cultural behaviours from an individual difference perspective and connects these interindividual differences back to larger personality systems that have been described across a wider range of cultures. Our results have demonstrated that it is possible to identify two dimensions of *jeitinho* which are linked to universal personality, value and attitude dimensions. Similar individual difference studies may uncover personality correlates of cultural behaviours in other contexts, for example, Hispanic *Simpatía* [26,45] or other informal social influence processes [5,6].

Focusing on the dimensionality, personal *jeitinho* comprises two dimensions that are theoretically consistent with the *jeitinho* concept described in previous anthropological, sociological and psychological work [1,2,7,8]. These two new dimensions represent the core features of the larger concept, including maintaining an affectionate relationship, as well as using trickery and cunningness to solve social problems.

The motivational base of *jeitinho* is coherent and in line with the predictions of the integrated model of personality motivations by Fischer [13]. Both personal *jeitinho* dimensions are rooted in a broad Behavioural Approach Motivation, but they differ in their orientation towards Trust-Based versus instrumental control motivations. Whereas *Jeitinho Simpatico* requires social interactions that grounded in mutual trust, *Jeitinho Malandro* is aimed at controlling and exploiting others. These broad dimensions are well-grounded in neuroscience, behavioural and evolutionary studies of individual differences in both humans and animals (for a general review, see [13]). Our main argument here is that basic human predispositions are expressed in culturally variant and specific behaviours, which nevertheless can still be traced back to broader psychological dimensions. Our study thereby contributes to an unpackaging of individual differences in a specific socio-cultural environment through the consideration of near-universal psychological dimensions. Cultural expressions of behaviour that is adaptive in a specific population and environments are based on basic predispositions that are shared through our common biological ancestry.

The present research has some limitations. First, our study relied on participants that were highly educated and had access to the internet. Since *jeitinho* is likely to vary across age, and educational level [2], this limits the generalizability of our findings to segments of Brazilian society that are less educated or have limited internet access. At the same time, the diversity of participants across our studies shows that the two major dimensions are replicable and robust. Given the similarity of behaviours such as *jeitinho* in other cultural settings [6], it might be interesting to examine how our two-dimensional structure operates in other cultural environments that are similarly characterized by high inequality, bureaucracy and economic stratification. Considering specifically the Big Five relationship with *jeitinho* we consider that further investigation should explore the correlations of *jeitinho* with the specific facets within the Big Five, in order to provide greater nuances and detail about how a behavioural trait such as *jeitinho* may fit with quasi-universal personality models. It is also important to explore the incremental validity of *jeitinho* compared to pan-cultural models of personality. To what extent do culture-specific traits such as *jeitinho* explain additional variance in relevant criterion variables, such as dishonest behaviour [33], or creativity [46] over and above established universal trait dimensions?

In summary, we believe we have made an important contribution to the study of personality and individual differences, by uncovering how culture-specific individual differences can be linked back to well-identified psychological processes. Our approach has the possibility to shed new light on the problem of comprehending the universality of personality structure by describing personality dimensions nested in indigenous socio-cultural processes. We hope new studies can shed further light on this issue, relating specific cultural patterns of social relationship personality dimensions to broader cross-cultural personality models.

## Supporting information

S1 File Supplemental material. All scales and items. Items of Personal *Jeitinho* Scale (PJS) are in Portuguese and English. All other scales are only in Portuguese.
(DOCX)
