# Supplementary material for: Between simpatia and malandragem: Brazilian jeitinho as an individual difference variable
Source: PLoS One. 2019 Apr 15;14(4):e0214929. doi: 10.1371/journal.pone.0214929 (PMC6464182; doi:10.1371/journal.pone.0214929)
Supplement: S1 File — All scales and items. Items of Personal Jeitinho Scale (PJS) are in Portuguese and English. All other scales are only in Portuguese. (DOCX) [file pone.0214929.s001.docx]

**SUPLEMENTAL MATERIALS: MEASURES ORIGINAL VERSIONS**

***PERNSONAL JEITINHO SCALE (PJS)***

**NOTE:** English version items are below the Portuguese version, if there is an interest to use the English translation, please contact the authors since there are some nuances in the Portuguese version that are not captured by the English translation.

**INSTRUÇÕES**. Nesta parte do questionário, apresentamos afirmações que descrevem algumas pessoas. Leia cada descrição e avalie o quanto cada uma dessas pessoas é semelhante a você. Para tal, utilize a escala abaixo que descreve opiniões que vão desde "Não se parece nada comigo" até "Se parece muito comigo".

| Não se parece nada comigo | Não se parece comigo | Se parece pouco comigo | Se parece mais ou menos comigo | Se parece comigo | Se parece muito comigo |
| --- | --- | --- | --- | --- | --- |
| **1** | **2** | **3** | **4** | **5** | **6** |

| Ele(a) gosta de manter o clima social agradável.  She/He likes to keep a pleasing social climate. | 1 2 3 4 5 6 |
| --- | --- |
| Ele(a) joga lixo no chão.  She/He throws trash on the floor. | 1 2 3 4 5 6 |
| Ele(a) entra em uma festa sem pagar por conhecer o produtor desta.  She/He get in a party without fee because she/he knows the party productor. | 1 2 3 4 5 6 |
| Ele(a) anda sem o cinto de segurança quando faz caminhos curtos  She/He uses a car without seat belt in short distances. | 1 2 3 4 5 6 |
| Ele(a) busca novos empreendimentos para sua profissão.  She/He seeks new enterprises to her/his career. | 1 2 3 4 5 6 |
| Ele(a) sempre dá alternativas, antes não pensadas, para solucionar os problemas dos amigos.  She/He always provides alternatives, not yet thought, to figure out friend’s problems. | 1 2 3 4 5 6 |
| Ele(a) passa no sinal vermelho quando a rua está vazia e sem pardal.  She/He crosses the red light of traffic light when the street is empty and without traffic radar. | 1 2 3 4 5 6 |
| Ele(a) cumprimenta a desconhecidos quando caminha pela rua.  She/He greets unknown people when she/he walks on the street. | 1 2 3 4 5 6 |
| Ele(a) estaciona na vaga de deficiente quando o estacionamento está cheio e precisa fazer algo rápido.  She/He parks in the disabled parking slot when the parking is full, and she/he is in a hurry. | 1 2 3 4 5 6 |
| Ele(a) se mostra bastante criativa ao enfrentar problemas no trabalho.  She/He shows herself/himself creative to deal with work challenges. | 1 2 3 4 5 6 |
| Ele(a) estaciona o carro em fila dupla, obstruindo a saída de outro veículo.  She/He parks in double queue obstructing other vehicles. | 1 2 3 4 5 6 |
| Ele(a) pede diferentes pratos ao retornar ao mesmo restaurante.  She/He asks different dishes when returns in the same restaurant. | 1 2 3 4 5 6 |
| Ele(a) cede a casa para churrascos da empresa.  She/He lends her/his house to company celebrations. | 1 2 3 4 5 6 |
| Ele(a) procura um conhecido que trabalha no cartório para adiantar seu processo.  She/He looks for an acquaintance that is notary’s office employee to advance her/his request. | 1 2 3 4 5 6 |
| Ele(a) mente em prol de um objetivo.  She/He lies towards a goal. | 1 2 3 4 5 6 |
| Ele(a) inventa novas receitas quando tem poucas opções de comida em casa.  She/He invents new recipes when she/he has few food choices. | 1 2 3 4 5 6 |
| Ele(a) segue o princípio: “Regras foram feitas para serem quebradas”.  She/He follows the principle: ‘Rules were done to be broken’. | 1 2 3 4 5 6 |
| Ele(a) segura a porta quando outra pessoa se aproxima.  She/He holds the door when someone else get closer. | 1 2 3 4 5 6 |
| Ele(a) está cansada na segunda-feira e liga no trabalho falando que está doente.  She/He is tired on Monday and calls to her/his job saying is sick. | 1 2 3 4 5 6 |
| Ele(a) mede as palavras para evitar conflitos.  She/He measures the words to avoid conflicts. | 1 2 3 4 5 6 |
| Ele(a), sabendo que certa pessoa ligará em determinado horário, desliga o celular e diz que estava sem bateria.  She/He, knowing that somebody will call in a specific time, turns off the mobile phone and says that the battery was over. | 1 2 3 4 5 6 |
| Ele(a) oferece ajuda aos colegas de trabalho.  She/He offers help to coworkers. | 1 2 3 4 5 6 |
| Ele(a) conversa durante uma sessão de cinema.  She/He chats during a movie session. | 1 2 3 4 5 6 |
| Ele(a), por vezes, não paga o condomínio no prazo determinado.  She/He frequently doesn’t pay the condominium fee in the right time. | 1 2 3 4 5 6 |
| Ele(a) sai da mesa do bar sem pagar a sua parte na conta quando esta deveria ser dividida igualmente por todos.  She/He leaves the bar table without paying her/his part of the bill when everybody should share it.  She/He enters in a party without fee due to knowing the promoter. | 1 2 3 4 5 6 |
| As pessoas se sentem queridas perto dele(a).  People feel desired near of her/him. | 1 2 3 4 5 6 |
| Ele(a) fala sobre as falhas de seus concorrentes a promoção no trabalho quando conversa com seus superiores.  She/He speaks about fails of competitor coworkers when chat with her/his superiors. | 1 2 3 4 5 6 |
| Ele(a) sempre cumprimenta o porteiro do seu prédio pelo nome toda vez que passa por ele na portaria.  She/He always greets by name the doorman of her/his building every time she/he enter by concierge. | 1 2 3 4 5 6 |
| Ele(a) quer comprar uma roupa para usar no final de semana, mas ao ver a loja fechando, convence a vendedora a vender-lhe.  She/He wants to buy a new clothes to use in the weekend, but when she/he realize the store is closing, convinces the saleswoman to sell. | 1 2 3 4 5 6 |
| Ele(a) faz o possível para devolver os livros na biblioteca antes do prazo.  She/He does the possible to return the books to the library before the deadline. | 1 2 3 4 5 6 |
| Ele(a) aproveita as oportunidades para prejudicar outras pessoas.  She/He seizes the opportunities to harm others. | 1 2 3 4 5 6 |

**PORTRAITS OF VALUES QUESTIONNAIRE**

**NOTE: English original version available here:** Schwartz, S. H., Melech, G., Lehmann, A., Burgess, S., Harris, M., & Owens, V. (2001). Extending the Cross-Cultural Validity of the Theory of Basic Human Values with a Different Method of Measurement. *Journal of Cross-Cultural Psychology*, *32*(5), 519–542. https://doi.org/10.1177/0022022101032005001

Descrevemos resumidamente abaixo algumas pessoas. Leia cada descrição e avalie o quanto cada uma dessas pessoas é semelhante a você. Assinale com um “X” a opção que indica o quanto a pessoa descrita se parece com você.

|  | Quanto esta pessoa se parece com você? |
| --- | --- |

|  | **Se parece muito comigo** | | **Se parece comigo** | **Se parece mais ou menos comigo** | | **Se parece pouco comigo** | **Não se parece comigo** | **Não se parece nada comigo** | |
| --- | --- | --- | --- | --- | --- | --- | --- | --- | --- |
| 1) Pensar em novas idéias e ser criativa é importante para ela. Ela gosta de fazer coisas de maneira própria e original. |  | |  |  | |  |  |  | |
| 2) Ser rica é importante para ela. Ela quer ter muito dinheiro e possuir coisas caras. |  | |  |  | |  |  |  | |
| 3) Ela acredita que é importante que todas as pessoas do mundo sejam tratadas com igualdade. Ela acredita que todos deveriam ter oportunidades iguais na vida. |  | |  |  | |  |  |  | |
| 4) É muito importante para ela demonstrar suas habilidades. Ela quer que as pessoas admirem o que ela faz. |  | |  |  | |  |  |  | |
| 5) É importante para ela viver em um ambiente seguro. Ela evita qualquer coisa que possa colocar sua segurança em perigo. |  | |  |  | |  |  |  | |
| 6) Ela acha que é importante fazer várias coisas diferentes na vida. Ela sempre procura novas coisas para experimentar. |  | |  |  | |  |  |  | |
| 7) Ela acredita que as pessoas deveriam fazer o que lhes é ordenado. Ela acredita que as pessoas deveriam sempre seguir as regras, mesmo quando ninguém está observando. |  | |  |  | |  |  |  | |
| 8) É importante para ela ouvir as pessoas que são diferentes dela. Mesmo quando não concorda com elas, ainda quer entendê-las. |  | |  |  | |  |  |  | |
| 9) Ela acha que é importante não querer mais do que se tem. Ela acredita que as pessoas deveriam estar satisfeitas com o que têm. |  | |  |  | |  |  |  | |
| 10) Ela procura todas as oportunidades para se divertir. É importante para ela fazer coisas que lhe dão prazer. |  | |  |  | |  |  |  | |
| 11) É importante para ela tomar suas próprias decisões sobre o que faz. Ela gosta de ser livre para planejar e escolher suas atividades. |  | |  |  | |  |  |  | |
| 12) É muito importante para ela ajudar as pessoas ao seu redor. Ela quer cuidar do bem-estar delas. |  | |  |  | |  |  |  | |
| 13) Ser muito bem-sucedida é importante para ela. Ela gosta de impressionar as demais pessoas. |  | |  |  | |  |  |  | |
| 14) A segurança de seu país é muito importante para ela. Ela acha que o governo deve estar atento a ameaças de origem interna ou externa. |  | |  |  | |  |  |  | |
| 15) Ela gosta de se arriscar. Ela está sempre procurando aventuras. |  | |  |  | |  |  |  | |
| 16) É importante para ela se comportar sempre corretamente. Ela quer evitar fazer qualquer coisa que as pessoas possam achar errado. |  | |  |  | |  |  |  | |
| 17) É importante para ela estar no comando e dizer aos demais o que fazer. Ela quer que as pessoas façam o que manda. |  | |  |  | |  |  |  | |
| 18) É importante para ela ser fiel a seus amigos. Ela quer se dedicar às pessoas próximas de si. |  | |  |  | |  |  |  | |
| 19) Ela acredita firmemente que as pessoas deveriam preservar a natureza. Cuidar do meio ambiente é importante para ela. |  | |  |  | |  |  |  | |
| 20) Ser religiosa é importante para ela. Ela se esforça para seguir suas crenças religiosas. | |  |  |  |  | |  |  |  |
| 21) É importante para ela que as coisas estejam organizadas e limpas. Ela realmente não gosta que as coisas estejam bagunçadas. | |  |  |  |  | |  |  |  |
| 22) Ela acha que é importante demonstrar interesse pelas coisas. Ela gosta de ser curiosa e tentar entender todos os tipos de coisas. | |  |  |  |  | |  |  |  |
| 23) Ela acredita que todas as pessoas do mundo deveriam viver em harmonia. Promover a paz entre todos os grupos no mundo é importante para ela. | |  |  |  |  | |  |  |  |
| 24) Ela acha que é importante ser ambiciosa. Ela quer demonstrar o quanto é capaz. | |  |  |  |  | |  |  |  |
| 25) Ela acha que é melhor fazer as coisas de maneira tradicional. É importante para ela manter os costumes que aprendeu. | |  |  |  |  | |  |  |  |
| 26) Aproveitar os prazeres da vida é importante para ela. Ela gosta de se mimar. | |  |  |  |  | |  |  |  |
| 27) É importante para ela entender às necessidades dos outros. Ela tenta apoiar aqueles que conhece. | |  |  |  |  | |  |  |  |
| 28) Ela acredita que deve sempre respeitar seus pais e os mais velhos. É importante para ela ser obediente. | |  |  |  |  | |  |  |  |
| 29) Ela quer que todos sejam tratados de maneira justa, mesmo aqueles que não conhece. É importante para ela proteger os mais fracos na sociedade. | |  |  |  |  | |  |  |  |
| 30) Ela gosta de surpresas. É importante para ela ter uma vida emocionante. | |  |  |  |  | |  |  |  |
| 31) Ela se esforça para não ficar doente. Estar saudável é muito importante para ela. | |  |  |  |  | |  |  |  |
| 32) Progredir na vida é importante para ela. Ela se empenha em fazer melhor que os outros. | |  |  |  |  | |  |  |  |
| 33) Perdoar as pessoas que lhe fizeram mal é importante para ela. Ela tenta ver o que há de bom nelas e não ter rancor. | |  |  |  |  | |  |  |  |
| 34) É importante para ela ser independente. Ela gosta de contar consigo mesmo. | |  |  |  |  | |  |  |  |
| 35) Contar com um governo estável é importante para ela. Ela se preocupa com a preservação da ordem social. | |  |  |  |  | |  |  |  |
| 36) É importante para ela ser sempre educada com os outros. Ela tenta nunca incomodar ou irritar os outros. | |  |  |  |  | |  |  |  |
| 37) Ela realmente quer aproveitar a vida. Divertir-se é muito importante para ela. | |  |  |  |  | |  |  |  |
| 38) É importante para ela ser humilde e modesta. Ela tenta não chamar atenção para si. | |  |  |  |  | |  |  |  |
| 39) Ela sempre quer ser aquela a tomar decisões. Ela gosta de liderar. | |  |  |  |  | |  |  |  |
| 40) É importante para ela se adaptar e se ajustar à natureza. Ela acredita que as pessoas não deveriam modificar a natureza. | |  |  |  |  | |  |  |  |

# BIG-FIVE 20 ITEM VERSION

**NOTE: English original version available here:** Benet-Martínez, V., & John, O. P. (1998). Los Cinco Grandes across cultures and ethnic groups: Multitrait-multimethod analyses of the Big Five in Spanish and English. *Journal of Personality and Social Psychology*, *75*(3), 729–750. https://doi.org/10.1037/0022-3514.75.3.729

**INSTRUÇÕES.** A seguir encontram-se algumas características (afirmações) que podem ou não lhe dizer respeito. Por favor, escolha um dos números na escala abaixo que melhor expresse sua opinião em relação a você mesmo e anote no espaço ao lado de cada afirmação. Vale ressaltar que não existem respostas certas ou erradas. Utilize a seguinte escala de resposta:

| **1** | **2** | **3** | **4** | **5** |
| --- | --- | --- | --- | --- |
| **Discordo totalmente** | **Discordo** | **Nem concordo nem discordo** | **Concordo** | **Concordo Totalmente** |

**Eu me vejo como alguém que....**

01.____É conversador, comunicativo.

02.____É minucioso, detalhista no trabalho, no que faz.

03.____Insiste até concluir a tarefa ou o trabalho.

04.____Gosta de cooperar com os outros.

05.____É original, tem sempre novas idéias.

06.____É temperamental, muda de humor facilmente.

07.____É inventivo, criativo.

08.____É prestativo e ajuda os outros.

09.____É amável, tem consideração pelos outros.

10.____Faz as coisas com eficiência.

11.____É sociável, extrovertido.

12.____É cheio de energia.

13.____É um trabalhador de confiança.

14.____Tem uma imaginação fértil.

15.____Fica tenso com freqüência.

16.____Fica nervoso facilmente.

17.____Gera muito entusiasmo.

18.____Gosta de refletir, brincar com as idéias.

19.____Tem capacidade de perdoar, perdoa fácil.

20.____Preocupa-se muito com tudo.

# MORALLY DEBATABLE BEHAVIOR SCALE

**NOTE: English original version available here:** Vauclair, C.-M., & Fischer, R. (2011). Do cultural values predict individuals’ moral attitudes? A cross-cultural multilevel approach. *European Journal of Social Psychology*, *41*(5), 645–657. https://doi.org/10.1002/ejsp.794

**INSTRUÇÕES.** Em que medida as afirmativas podem ser justificadas, não podem ser justificadas ou alguma opinião entre essas duas. Na escala de 1 a 10, 1 significa “nunca se justifica” e 10 significa “sempre se justifica” . *Leia e assinale uma alternativa para cada item*.

| Nunca se justifica | | | | | Sempre se justifica | | | | |
| --- | --- | --- | --- | --- | --- | --- | --- | --- | --- |
| 1 | 2 | 3 | 4 | 5 | 6 | 7 | 8 | 9 | 10 |

|  | Nunca se  justifica | | | | | Sempre se  justifica | | | | |
| --- | --- | --- | --- | --- | --- | --- | --- | --- | --- | --- |
| Pedir benefícios do governo sem ter direito. | 1 | 2 | 3 | 4 | 5 | 6 | 7 | 8 | 9 | 10 |
| Evitar pagar passagem em transporte público. | 1 | 2 | 3 | 4 | 5 | 6 | 7 | 8 | 9 | 10 |
| Não pagar impostos se tiver a chance. | 1 | 2 | 3 | 4 | 5 | 6 | 7 | 8 | 9 | 10 |
| Aceitar suborno para cumprir seu dever. | 1 | 2 | 3 | 4 | 5 | 6 | 7 | 8 | 9 | 10 |

# SOCIAL DOMINANCE ORIENTATION

**NOTE: English original version available here:** Pratto, F., Sidanius, J., Stallworth, L. M., & Malle, B. F. (1994). Social dominance orientation: A personality variable predicting social and political attitudes. *Journal of Personality and Social Psychology*, *67*(4), 741–763. https://doi.org/10.1037/0022-3514.67.4.741

**INSTRUÇÕES.** Nesta parte do questionário, gostaríamos de saber qual o seu sentimento em relação às afirmações a seguir. Ao lado de cada declaração, assinale o número de 1 a 7 que represente o grau do seu sentimento positivo ou negativo em relação a cada frase.

| **1** | **2** | **3** | **4** | **5** | **6** | **7** |
| --- | --- | --- | --- | --- | --- | --- |
| Discordo totalmente | Discordo | Discordo  um pouco | Nem concordo nem discordo | Concordo  um pouco | Concordo | Concordo totalmente |

| Deveriam ser dadas chances iguais na vida a todos os grupos. | 1 | 2 | 3 | 4 | 5 | 6 | 7 |
| --- | --- | --- | --- | --- | --- | --- | --- |
| É certo que alguns grupos tenham mais chances na vida do que outros. | 1 | 2 | 3 | 4 | 5 | 6 | 7 |
| Grupos inferiores deveriam ficar nos seus lugares. | 1 | 2 | 3 | 4 | 5 | 6 | 7 |
| Nós deveríamos nos esforçar para que todos recebessem salários iguais. | 1 | 2 | 3 | 4 | 5 | 6 | 7 |
| Nós teríamos menos problemas se certos grupos ficassem nos seus lugares. | 1 | 2 | 3 | 4 | 5 | 6 | 7 |
| Seria bom se todos os grupos pudessem ser iguais. | 1 | 2 | 3 | 4 | 5 | 6 | 7 |

**BRAZILIAN JEITINHO QUESTIONNAIRE (BJQ)**

**NOTE: English version is available in this paper:** Ferreira, M. C., Fischer, R., Porto, J. B., Pilati, R., & Milfont, T. L. (2012). Unraveling the mystery of Brazilian jeitinho: a cultural exploration of social norms. *Personality & Social Psychology Bulletin*, *38*(3), 331–44. https://doi.org/10.1177/0146167211427148

**INSTRUÇÕES.** A seguir você encontrará uma série de situações corriqueiras. Sua tarefa consiste em ler atentamente cada uma das situações e responder qual a probabilidade de você se comportar como a pessoa descrita em cada uma delas. Para dar sua resposta utilize a seguinte escala:

| Muito improvável | **0** | **1** | **2** | **3** | **4** | **5** | **6** | **7** | **8** | **9** | **10** | Muito provável |
| --- | --- | --- | --- | --- | --- | --- | --- | --- | --- | --- | --- | --- |

Assim, se você avaliar que é **muito provável** você se comportar como a pessoa descrita, você deve marcar um X no número 10. Por outro lado, se você considera **muito improvável** se comportar como a pessoa descrita, marque um X no número 0. Varie suas respostas em função do quanto você acha provável se comportar como a pessoa descrita em cada uma das situações. Tente usar toda a extensão da escala de respostas.

| Carla não conseguiu se organizar ao longo do semestre e, na época de avaliações, há um conteúdo muito grande a ser estudado. Dias antes da prova, lembra que a ementa da disciplina inclui um livro muito grande, que não terá tempo de ler. Assim, antes da prova, Carla lê o resumo encontrado na internet, para ao menos se inteirar do conteúdo do livro. | | | | | | | | | | | | |
| --- | --- | --- | --- | --- | --- | --- | --- | --- | --- | --- | --- | --- |
| Muito improvável | **0** | **1** | **2** | **3** | **4** | **5** | **6** | **7** | **8** | **9** | **10** | Muito provável |
| Daniel é morador de rua e percebe que está cada vez mais difícil receber esmolas. Assim, para garantir sua subsistência, decide improvisar apresentações de malabarismo numa praça movimentada, para arrecadar dinheiro. | | | | | | | | | | | | |
| Muito improvável | **0** | **1** | **2** | **3** | **4** | **5** | **6** | **7** | **8** | **9** | **10** | Muito provável |
| É aniversário da mãe de Antônio e ele está sem dinheiro para comprar um presente para ela. A mãe dele gosta muito de flores perfumadas. Antônio decide, então, pegar uma flor do arranjo de flores de plástico de sua casa e dar de presente a sua mãe. Todavia, por ser de plástico, a flor não tem cheiro. Ele resolve esse problema colocando perfume na flor de plástico e presenteando sua mãe com a flor. | | | | | | | | | | | | |
| Muito improvável | **0** | **1** | **2** | **3** | **4** | **5** | **6** | **7** | **8** | **9** | **10** | Muito provável |
| É dia do aniversário de um grande amigo de Joana e ela se esqueceu de comprar o presente. Além disso, está com problemas financeiros e acredita que não irá encontrar nada acessível de última hora. Joana, que é muito habilidosa, separa alguns materiais de escola e faz um belo cartão para presentear seu amigo. | | | | | | | | | | | | |
| Muito improvável | **0** | **1** | **2** | **3** | **4** | **5** | **6** | **7** | **8** | **9** | **10** | Muito provável |
| Em uma empresa, quando a secretária do diretor falta, a funcionária Paula tem que assumir seu posto de trabalho. Contudo, Paula tem outros serviços para fazer. Se ela não fizer, o trabalho se acumula e outros setores da empresa se prejudicam. Para que isso não aconteça, Paula propõe a outra pessoa que fique no lugar da secretária do diretor, nos momentos em que ela está muito assoberbada. | | | | | | | | | | | | |
| Muito improvável | **0** | **1** | **2** | **3** | **4** | **5** | **6** | **7** | **8** | **9** | **10** | Muito provável |
| Flávio trabalha como corretor de imóveis em uma imobiliária. Às vezes, quando vê que o proprietário está pedindo um valor abaixo do valor de mercado, ele consegue cobrar um valor maior do comprador, sem constar do papel e, com isso, ganhar um dinheiro a mais por fora. | | | | | | | | | | | | |
| Muito improvável | **0** | **1** | **2** | **3** | **4** | **5** | **6** | **7** | **8** | **9** | **10** | Muito provável |
| José, todas as vezes que toma taxis por motivos de trabalho, tem direito a pedir ressarcimento dos valores pagos. Quando está muito sem dinheiro, ele pega uma conta de valor maior que o real e apresenta no trabalho, de modo a ficar com um ganho extra. | | | | | | | | | | | | |
| Muito improvável | **0** | **1** | **2** | **3** | **4** | **5** | **6** | **7** | **8** | **9** | **10** | Muito provável |
| Josué conheceu um novo restaurante em sua cidade. Ele gostou muito do estabelecimento e, para evitar esperar na fila nos dias que vai ao restaurante, decide dar boas gorjetas ao garçom, para que não precise esperar na fila. | | | | | | | | | | | | |
| Muito improvável | **0** | **1** | **2** | **3** | **4** | **5** | **6** | **7** | **8** | **9** | **10** | Muito provável |
| Manuel é funcionário de confiança de uma empresa, sendo responsável pelos pagamentos da mesma. Como ele está todo endividado, aproveita-se de uma brecha e desvia um dinheiro extra para sua própria conta-corrente, sem que ninguém perceba, conseguindo, assim, respirar um pouco mais aliviado em relação a suas dívidas. | | | | | | | | | | | | |
| Muito improvável | **0** | **1** | **2** | **3** | **4** | **5** | **6** | **7** | **8** | **9** | **10** | Muito provável |
| Maria tinha uma reunião de trabalho programada para 8h. Todavia, ela se atrasou para ir até o local da reunião. Sabendo que não conseguiria chegar a tempo, e por não ter um motivo suficientemente forte para justificar a situação, ela liga dizendo que teve problemas com seu carro, mas que, apesar disso, já está se dirigindo ao local da reunião. | | | | | | | | | | | | |
| Muito improvável | **0** | **1** | **2** | **3** | **4** | **5** | **6** | **7** | **8** | **9** | **10** | Muito provável |
| Marília trabalha como auxiliar de serviços gerais em uma grande empresa e não consegue renda suficiente para pagar as contas de sua casa. Então, para complementar a renda, ela conversa com seu chefe e pede autorização para vender sanduíches e salgados para os funcionários da empresa em que trabalha. | | | | | | | | | | | | |
| Muito improvável | **0** | **1** | **2** | **3** | **4** | **5** | **6** | **7** | **8** | **9** | **10** | Muito provável |
| Marina precisa ir rapidamente ao mercado comprar somente um litro de leite para fazer a mamadeira de seus filhos. Ao chegar ao mercado, porém, verifica que não há vagas para estacionar o carro. Ela pára então o carro em cima da calçada, liga o pisca-alerta para disfarçar, e entra rápido no mercado para comprar o leite. | | | | | | | | | | | | |
| Muito improvável | **0** | **1** | **2** | **3** | **4** | **5** | **6** | **7** | **8** | **9** | **10** | Muito provável |
| O professor de João havia passado para seus alunos um exercício a ser entregue na aula seguinte, como parte da avaliação da disciplina. João, porém, não conseguiu se organizar para finalizá-lo na aula programada. Assim, o aluno deixou de ir à aula e entregou a tarefa na aula seguinte, argumentando que não pôde comparecer à aula anterior. | | | | | | | | | | | | |
| Muito improvável | **0** | **1** | **2** | **3** | **4** | **5** | **6** | **7** | **8** | **9** | **10** | Muito provável |
| Os estacionamentos de shoppings ficam muito cheios em datas próximas a festividades. Sabendo que é muito difícil encontrar vagas nesse período, Camila conversa com sua avó e a convida para acompanhá-la às compras, para poder estacionar na vaga reservada a idosos. | | | | | | | | | | | | |
| Muito improvável | **0** | **1** | **2** | **3** | **4** | **5** | **6** | **7** | **8** | **9** | **10** | Muito provável |
| Os ingressos para o último show da turnê do artista preferido de Eduardo já estão se esgotando e, ao chegar ao local de venda, ele vê que há uma fila muito grande e que dificilmente conseguirá comprar os ingressos. Logo no começo dessa fila, porém, ele encontra um colega de escola que já não via há algum tempo. Como Eduardo tem realmente muita vontade de ir a esse show, decide cumprimentar o amigo e pede para ficar com ele na fila. | | | | | | | | | | | | |
| Muito improvável | **0** | **1** | **2** | **3** | **4** | **5** | **6** | **7** | **8** | **9** | **10** | Muito provável |
| Pablo está com a lanterna do carro quebrada. Dirigindo em uma estrada à noite, é parado por um policial rodoviário. Para não ser multado, Pablo oferece um dinheiro ao policial para que ele lhe deixe prosseguir viagem. | | | | | | | | | | | | |
| Muito improvável | **0** | **1** | **2** | **3** | **4** | **5** | **6** | **7** | **8** | **9** | **10** | Muito provável |
| Paulo bate com seu carro e não tem seguro contra colisões. Para não ter um prejuízo muito grande, ele consegue fazer um seguro com data anterior ao acidente, de modo a que a seguradora possa cobrir os danos provocados pelo acidente. | | | | | | | | | | | | |
| Muito improvável | **0** | **1** | **2** | **3** | **4** | **5** | **6** | **7** | **8** | **9** | **10** | Muito provável |
| Tampinha ganha a vida como motoboy. Para complementar sua renda, trabalha como garçom performático em um restaurante todas as noites, esbanjando simpatia. Com seu desprendimento, consegue conquistar toda a clientela. | | | | | | | | | | | | |
| Muito improvável | **0** | **1** | **2** | **3** | **4** | **5** | **6** | **7** | **8** | **9** | **10** | Muito provável |
| Um vereador, muito conhecido em sua cidade, consegue obter ajuda em material de construção para a reforma de uma escola, com os construtores da região. Ele, porém, pega parte desse material e usa na reforma da casa de um de seus filhos. | | | | | | | | | | | | |
| Muito improvável | **0** | **1** | **2** | **3** | **4** | **5** | **6** | **7** | **8** | **9** | **10** | Muito provável |
